# Assembly of Inflammation-Related Genes for Pathway-Focused Genetic Analysis

**DOI:** 10.1371/journal.pone.0001035

**Published:** 2007-10-17

**Authors:** Matthew J. Loza, Charles E. McCall, Liwu Li, William B. Isaacs, Jianfeng Xu, Bao-Li Chang

**Affiliations:** 1 Center for Human Genomics, Department of Internal Medicine, Wake Forest University School of Medicine, Winston-Salem, North Carolina, United States of America; 2 Department of Internal Medicine, Wake Forest University School of Medicine, Winston-Salem, North Carolina, United States of America; 3 Department of Biology, Virginia Polytechnic Institute and State University, Blacksburg, Virginia, United States of America; 4 Department of Urology, Johns Hopkins University Medical Institutions, Baltimore, Maryland, United States of America; 5 Department of Oncology, Johns Hopkins University Medical Institutions, Baltimore, Maryland, United States of America; 6 Center for Human Genomics, Department of Epidemiology and Prevention, Wake Forest University School of Medicine, Winston-Salem, North Carolina, United States of America; 7 Center for Human Genomics, Department of Pediatric Medicine, Wake Forest University School of Medicine, Winston-Salem, North Carolina, United States of America; Oregon Health & Science University, United States of America

## Abstract

Recent identifications of associations between novel variants in inflammation-related genes and several common diseases emphasize the need for systematic evaluations of these genes in disease susceptibility. Considering that many genes are involved in the complex inflammation responses and many genetic variants in these genes have the potential to alter the functions and expression of these genes, we assembled a list of key inflammation-related genes to facilitate the identification of genetic associations of diseases with an inflammation-related etiology. We first reviewed various phases of inflammation responses, including the development of immune cells, sensing of danger, influx of cells to sites of insult, activation and functional responses of immune and non-immune cells, and resolution of the immune response. Assisted by the Ingenuity Pathway Analysis, we then identified 17 functional sub-pathways that are involved in one or multiple phases. This organization would greatly increase the chance of detecting gene-gene interactions by hierarchical clustering of genes with their functional closeness in a pathway. Finally, as an example application, we have developed tagging single nucleotide polymorphism (tSNP) arrays for populations of European and African descent to capture all the common variants of these key inflammation-related genes. Assays of these tSNPs have been designed and assembled into two Affymetrix ParAllele customized chips, one each for European (12,011 SNPs) and African (21,542 SNPs) populations. These tSNPs have greater coverage for these inflammation-related genes compared to the existing genome-wide arrays, particularly in the African population. These tSNP arrays can facilitate systematic evaluation of inflammation pathways in disease susceptibility. For additional applications, other genotyping platforms could also be employed. For existing genome-wide association data, this list of key inflammation-related genes and associated subpathways can facilitate comprehensive inflammation pathway- focused association analyses.

## Introduction

Inflammation is an essential component of immune-mediated protection against pathogens and tissue damage. Immune responses are also responsible for the unfavorable rejection of tissue/organ transplants, hypersensitivity reactions (e.g., atopy, anaphylaxis, contact hypersensitivity, delayed-type hypersensitivity), and septic shock. Aberrant or unchecked immune responses may lead to a state of chronic inflammation [1]–[3]. This may occur when the immune response: 1) is activated in the absence of ‘danger’ signals; 2) fails to fully turn-off (resolve) after elimination of the danger; and 3) fails to completely clear the danger stimulus. Factors that may influence the initiation, activity, and resolution of immune responses include health (physical and emotional), age, diet, medications, and genetic predisposition.

Inflammation may also be a contributing factor for some diseases. The role of chronic inflammation in a wide variety of diseases is well-appreciated, including rheumatoid arthritis and other autoimmune disorders [4], cardiovascular disease [5]–[7], gastrointestinal disorders [8], [9], and a number of cancers [10]–[14]. Perhaps the best evidence for the importance of chronic inflammation in disease is the efficacy of NSAIDs in reducing the risk or severity of these disorders [15]. There is mounting evidence that dietary factors that may influence inflammation, such as the balance of omega-3 vs. omega-6 polyunsaturated fatty acids (PUFAs), have an impact on disease risk and progression [16]. Genetic studies also provide evidence that variant alleles of genes associated with inflammatory pathways impact the risk of disease initiation, progression, and severity (see **Table 1**). The role of inflammation as a mediator of disease is currently receiving extensive attention, resulting in the National Institute of Allergy and Immunologic Diseases (NIAID) plans for an NIH Roadmap initiative with the overarching theme: “Inflammation as a Common Mechanism of Disease” (http://nihroadmap.nih.gov/inflammation/index.asp).

**Table 1:** Confirmed associations of genetic variants in inflammation-associated genes and disease.

Disease	Gene	Encoded protein	Variant	Odds ratio a	p-value	Confirmation method
Age-related macular degeneration [44]	*CFH*	Complement factor H	rs1061170	3.40^b^	<1×10^−5^	Case-control/meta-analysis
Atopic asthma [32]	*IL4R*	IL-4 receptor alpha	rs1801275	1.79	3×10^−9^	Meta-analysis of 7 study populations
Atopic asthma [21]	*TNF*	TNF-alpha	-308 G/A	1.46	1×10^−4^	Meta-analysis of 15 study populations
Crohn's disease [30]	*CARD15*	Nod2	1007fsinsC	4.3	7×10^−28^	Meta-analysis of 16 study populations
Breast cancer [33]	*CASP8*	Caspase-8	rs1045485	0.90	0.016	Analysis of 3 study populations (6351 cases/5708 controls)
Breast cancer [33]	*TGFB1*	TGF-beta 1	rs1982073	1.08	0.0088	Analysis of 3 study populations (6863 cases/5587 controls)
Breast cancer [34]	*TNF*	TNF-alpha	rs361525	1.18	0.008	Analysis of two independent study populations
Graves' disease [24]	*PTPN22*	Lymphoid-specific phosphatase	C1858T	1.61	<1×10^−5^	Meta-analysis of 3 study populations
Inflammatory bowel disease [31]	*IL23R*	IL-23 receptor beta	rs11209026	0.26	5×10^−9^	Genome-wide screen (raw p-value)
				0.45	8×10^−4^	Case-control replication
				∼0.5 c	1.3×10^−10^	Family-based TDT replication
Psoriatic arthritis [23]	*TNF*	TNF-alpha	-238 G/A	2.29	2×10^−4^	Meta-analysis of 8 study populations
Rheumatoid arthritis [24]	*PTPN22*	Lymphoid-specific phosphatase	C1858T (R620W)	1.68	<1×10^−5^	Meta-analysis of 12 study populations
Systemic lupus erythamatosus [25]	*IRF5*	Interferon response factor 5	rs2004640	1.47	4.2×10^−21^	Case-control/meta-analysis+replication in family-based
Systemic lupus erythamatosus [24]	*PTPN22*	Lymphoid-specific phosphatase	C1858T (R620W)	1.49	<1×10^−5^	Meta-analysis of 5 study populations
Systemic lupus erythamatosus [22]	*TNF*	TNF-alpha	-308 G/A	2.1	<0.001	Meta-analysis of 10 study populations of European descent
Type 1 diabetes [29]	*CTLA4*	CTLA-4	rs3087243	1.18 c	5.6×10^−6^	Family-based TDT
Type 1 diabetes [28]	*CTLA4*	CTLA-4	rs3087243	1.17 c	6×10^−4^	Family-based TDT
				1.21	1.3×10^−7^	Case-control
Type 1 diabetes [27]	*CTLA4*	CTLA-4	rs3087243	1.20	3.7×10^−10^	Case-control
Type 1 diabetes [26]	*IFIH1*	Mda-5, Helicard	rs1990760	0.86	1.42×10^−10^	Genome-wide, validated in case-control+family-based
Type 1 diabetes [24]	*PTPN22*	Lymphoid-specific phosphatase	C1858T (R620W)	1.85	<1×10^−5^	Meta-analysis of 6 study populations

a Odds ratio for allele test (multiplicative model), unless otherwise indicated.

b Odds ratio for dominant model

c Risk ratio from family-based transmission disequilibrium test (TDT).

Numerous genetic linkage and case-control association studies have implicated genetic variations in genes important in immunity and inflammation and inflammatory diseases. Single missense heritable mutations can be the sole or major determinant for inflammatory diseases, such as Familial Cold Autoinflammatory Syndrome (missense mutations in exon 3 of *Cias1* account for all cases) [17]–[19] and Familial Mediterranean Fever (*MEFV*, five founder missense mutations, when homozygous, account for 74% of cases) [20]. For many complex inflammatory diseases, polymorphisms in inflammatory genes are more likely to act as modifiers for disease susceptibility rather than sole determinants. Recent meta-analyses report modest associations between single nucleotide polymorphisms (SNPs) in *TNF* (encoding TNF-α) and increased risk for asthma [21], system lupus erythamatosus (SLE) [22], and psoriatic arthritis [23]. A variant of *PTPN22* (encoding a lymphoid-specific protein tyrosine phosphatase) is modestly associated with multiple autoimmune diseases (rheumatoid arthritis, SLE, type 1 diabetes, and Graves' disease) [24]. Associations of *IRF5* (interferon regulator factor 5) genetic variants and increased SLE risk have been highly replicated [25]. *IFIH1*, encoding an innate immunity viral mRNA detector (early type I IFN-β responsive gene, Helicard), is strongly associated with type 1 diabetes risk [26]. Association of a small risk for type 1 diabetes and *CTLA4* has been consistently replicated [27]–[29]. An insertion polymorphism in *CARD15*, the gene encoding the microbial nucleotide detector Nod2, is a major risk factor for Crohn's disease [30]. An association between SNP's in *IL23R* (IL-23 receptor β-chain) and increased risk of inflammatory bowel disease has been reported for a genome-wide association study and confirmed in three independent populations [31]. A recent meta-analysis demonstrated that an *IL4R* (IL-4 receptor alpha chain) variant modestly increases risk for atopic asthma [32]. A description of these studies and reported associations is provided in **Table 1**. This list is not intended to be comprehensive but rather serves as an example of representative genetic variations which have been well-established to be associated with common inflammatory disorders.

In addition to inflammatory/autoimmune diseases, polymorphisms in inflammation-associated genes may also contribute to risk for diseases in which inflammatory/immune-disorders are not the primary characteristic. There is evidence from the Breast Cancer Association Consortium that *CASP8* (caspase 8) and *TGFB1* (TGF-β1) variants impart risk, albeit low penetrance, for breast cancer [33]. Analysis of two independent populations suggest that a rare polymorphism in *TNF* may also be a low-penetrance risk factor for breast cancer [34]. Several groups report associations between various *PTGS2* (COX-2) genetic variants and colorectal cancer risk [35]–[39]. There is also evidence to suggest that a low frequency *COX2* variant allele decreases prostate cancer risk [40], particularly in subjects who frequently eat fish [41]. Interestingly, in a preliminary study using MegAllele™ I&I panel, including 9,275 SNPs in 1,086 genes involved in immunity and inflammation, more SNPs were found to be significantly associated with prostate cancer risk than expected by chance, which suggests multiple genetic variants in this pathway impart modest risk to prostate cancer [42].

Because genetic associations of disease and genetic variations in inflammatory genes are often relatively modest, it is likely that polymorphisms in multiple inflammatory genes cooperate in an additive or synergistic manner to impact disease risk. Pathway analyses may help to reveal gene-gene interactions or risks imparted independently from other genes in the pathway. The advantages of performing analyses at pathway levels are illustrated by Dinu et al. [43]. Associations between *CFH* (complement factor H) and age-related macular degeneration have been replicated in numerous studies [44]. To test whether genetic variations in the multiple complement pathway genes impact macular degeneration risk, Dinu et al. analyzed the existing genome-wide association study data of Klein et al. [45], restricting the analyses to genes in the complement pathway in subjects carrying the *CFH* risk allele. Significant associations were detected for a *C7* and *MBL2* variants and severity of macular degeneration in the context of the complement pathway analysis and the *CHF* risk allele, and these associations would not have been significant in a genome-wide analysis of the data.

## Results

### Choosing genes

**Figure 1:**
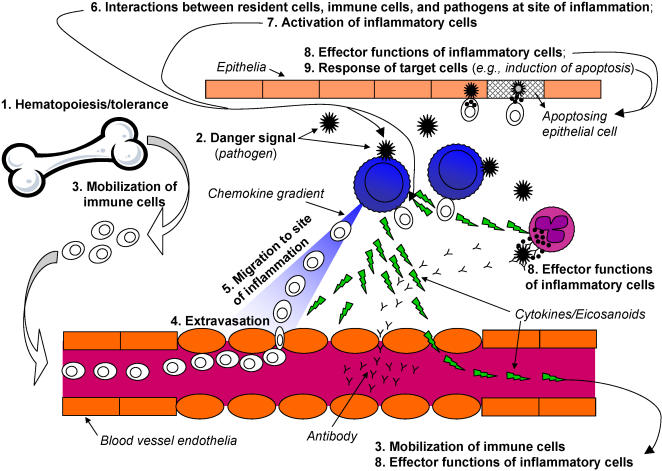
Development of an immune response. Depicted is a schematic representation of an immune response to a generic pathogenic insult. The phases of immune responses (described in Table 2) are shown in bold. Additional aspects not shown are the involvement of secondary lymphoid tissues for initial T cell and B cell activation by dendritic cells that migrate from the site of inflammation to lymph nodes and other secondary lymphoid structures. The resolution of immune responses, immunological memory, and homeostasis are also not depicted.

Various aspects of immunity contribute to the development of an overall inflammatory immune response. These phases include the development of immune cells, sensing of danger, influx of cells to sites of insult, activation and functional responses of immune and non-immune cells, and resolution of the immune response. To broadly cover most aspects of inflammatory responses, the various *phases of immune responses* were considered in choosing genes for the SNP array panel, outlined in **Table 2**. A schematic representation of most of these phases is presented in **Fig. 1**.

**Table 2:** Phases of immune response.

Phase of immune response	Description
Hematopoiesis/homeosta-sis/tolerance	The generation and differentiation of immune cells and maintenance of their number in circulation and tissues; prevention of self-reactivity.
Danger signal	Innate recognition of and response to pathogenic foreign substances or stress.
Mobilization of immune cells	Systemic soluble mediators informing immune cells in circulation and lymphoid tissues of danger.
Extravasation	The process of circulating immune cells crossing from blood into peripheral tissues and secondary lymphoid tissues.
Migration to site of inflammation	The process of immune cells, after extravasation, reaching the site of inflammatory insult, including chemoattraction, adhesion to substrates, and degradation of extracellular matrix.
Interactions between resident cells, immune cells, and pathogens at site of inflammation	Interactions between resident cells, immune cells, and pathogens at site of inflammation–how infiltrating cells interact with the resident inflammatory cells, non-immune cells (e.g., epithelia), pathogens, and other infiltrating cells, that leads to activation of effector functions.
Activation of inflammatory cells	The signaling pathways and transcription factors stimulated by activating, co-stimulatory, and inhibitory receptors that leads to activation, proliferation, differentiation, and survival of responding immune cells.
Effector functions of inflammatory cells	The factors produced/released by immune cells in attempt to resolve the pathogenic insults, including release of cytotoxic/cytostatic mediators and mediators to enhance or fine-tune the immune response.
Response of target cells	The pathways in non-immune cells (e.g., epithelia) activated in response to the effector functions of immune cells.
Resolution of immune response vs. chronic inflammation	The pathways that lead to the downregulation of immune responses and inflammation after the pathogenic insult is cleared; the factors maintaining late-phase immune responses when the insult is not totally resolved.

Priority was given first to genes of known function in inflammatory responses (in both immune and non-immune cells), and then to genes expressed in immune cells with function implied by homology to other genes but exact function not clear. Ubiquitously expressed genes required for the normal function of most cell types of diverse origin were given lower priority. However, special emphasis was placed on genes at nodes for signaling to and from multiple pathways, most notably genes in NF-κB, MAPK, and PI3K signaling pathways.

Pathways were built using Ingenuity Pathways Analysis, as described in *Methods* section, using both pre-defined ‘canonical pathways’ and custom-built pathways based on our own queries for genes/pathways not included in the canonical pathways.

**Figure 2:**
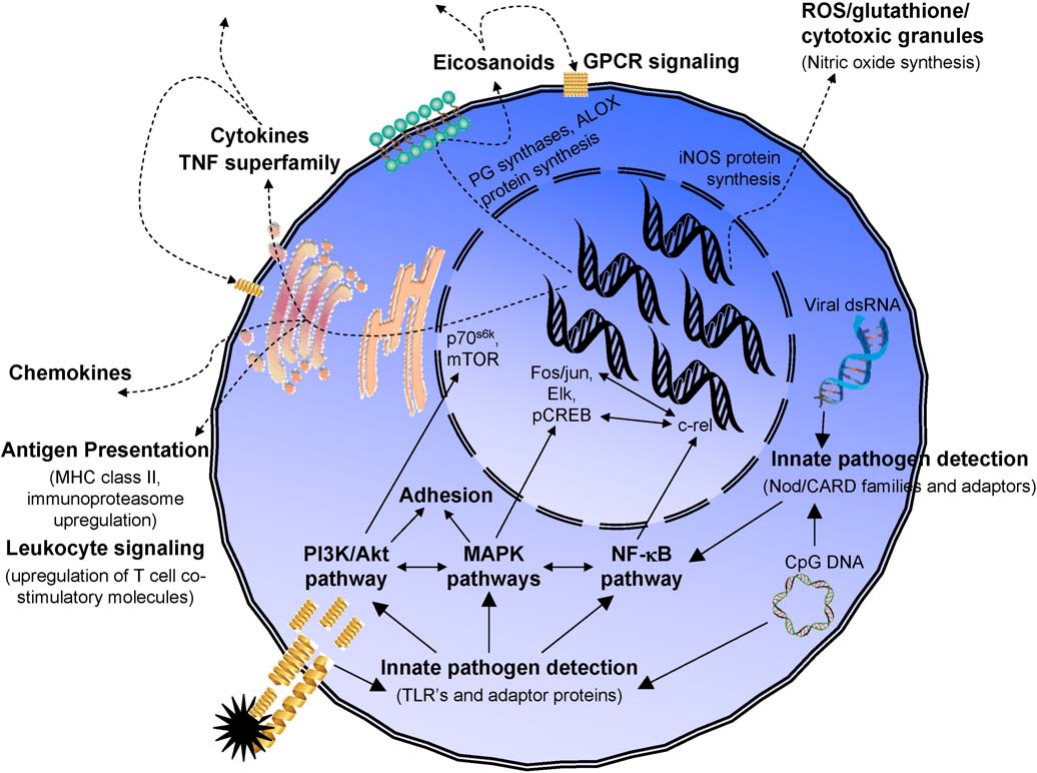
Inflammation subpathways involved in the response to danger signal. The concerted action of multiple functional subpathways in the initial response of a macrophage to bacteria or virus is depicted. Solid arrows indicate signaling events and dashed arrows stimulated production of proteins and other inflammatory mediators (including autocrine/paracrine responses of the macrophage to the released molecules).

Multiple functional pathways are involved each of the immune response phases, and each functional pathway may contribute to several of the immune response phases. For example (**Fig. 2**), the response of a macrophage responding to a *danger signal* during a gram-negative bacterial infection involves innate pathogen recognition of LPS by the TLR4 complex. TLR4 transduces signals via NF-κB, MAPK, and PI3K signaling pathways, stimulating synthesis of eicosanoids and cytokines to signal other cells of the danger. LPS may also stimulate expression of stress-induced proteins, such as MIC-A and MIC-B (ligands for natural killer cell activating receptors) and T cell co-stimulatory molecules (B7 family proteins).

Because immune response phases utilize multiple functional pathways and these pathways are overlapping among phases, the genes chosen for the SNP array panel were assigned to one of the following *functional pathways*:

**Adhesion-Extravasation-Migration:** adhesion molecules; chemoattractants and chemoattractant receptors; cytoskeletal rearrangement signaling, motility proteins.

**Apoptosis signaling:** death receptors and ligands and extrinsic apoptosis pathway signaling; mitochondrial-dependent, intrinsic apoptosis pathway signaling; cellular stress signaling.

**Calcium signaling:** NF-ATs; calcineurins; calcium/calmodulin-dependent kinases.

**Complement cascade:** components of classical, alternative, and lectin-dependent complement pathways.

**Cytokines and cytokine signaling:** cytokines; cytokine receptors; cytokine-dependent signaling, including JAK-STAT and interferon-regulatory factor (IRF) pathways; suppressor of cytokine signaling proteins (SOCS).

**Eicosanoid synthesis and receptors:** enzymes involved in synthesis of prostanoids, leukotrienes, hepoxylins (12-HETE), and lipoxins from arachidonic acid; prostanoid and leukotriene receptors.

**Glucocorticoid/PPAR signaling:** nuclear receptors for glucocorticoids; steroid-interacting proteins; PPARs and associated proteins.

**G-Protein Coupled Receptor Signaling:** GPCRs (other than eicosanoid receptors and chemokine receptors); G-protein-dependent signaling pathways, including cAMP-PKA and phospholipase B2.

**Innate pathogen detection:** Toll-like receptors (TLRs); intracellular nucleotide detectors (e.g., Nod1 and putative members of CARD/Nod family); peptidoglycan recognition proteins; associated signaling molecules linking these detectors to major signaling pathways.

**Leukocyte signaling:** Signaling molecules, receptors, and adaptors important for regulation of leukocyte activation beyond major signaling pathways (i.e., MAPK, PI3K/Akt, NF-κB, GPCR, and cytokine signaling pathways); including, but not limited to, T cell receptor (TCR) and B cell receptor (BCR) signaling components, B7 family, phosphatases, Foxp3, immunoglobulin receptors, leukocyte inhibitory receptor (CD85) family, scavenger receptors.

**MAPK signaling:** p38 stress-activated protein kinase, p42/p44 extracellular-regulated kinase (Erk), and Jun kinase (Jnk) signaling pathways.

**Natural Killer Cell Signaling:** Natural killer (NK) cell-specific activating and inhibitory receptors; adaptors and signaling molecules for transducing NK cell-specific receptor signaling.

**NF-kB signaling:** Molecules for regulation of NF-κB activation, including adaptors linking. other pathways to and from the NF-κB pathway (e.g., from MAPK, pathogen-detection, and TNF-α signaling pathways).

**Antigen presentation:** Major Histocompatibility Complex (MHC) molecules and associated proteins; proteins involved in uptake, processing, and loading of peptides on MHC molecules.

**PI3K/AKT Signaling:** Molecules involved in regulation of PI3K-dependent signaling, including adaptors linking other pathways to and from the PI3K pathway.

**ROS/glutathione/cytotoxic granules:** Molecules involved in the generation and response to leukocyte-derived cytotoxic agents (reactive oxygen species (ROS), nitric oxide, cytotoxic granules of granulocytes and natural killer cells), including contents of granulocyte and NK cell cytotoxic granules; glutathione peroxidases; peroxiredoxins; catalase; proteinases; superoxide dismutase.

**TNF superfamily and signaling:** Receptors and ligands of the TNF-α superfamily; adaptors and signaling molecules involved in transducing signals from receptor stimulation to other major signaling pathways (e.g., MAPK and NF-κB pathways).

Examples of the functional subpathways and types of genes chosen for the different phases of immune responses are presented in **Table 3** (proteins encoded by the genes are provided). Most of these immune response phases also utilize common signaling pathways, including elements of MAPK, NF-κB, PI3K/Akt, GCPR, cytokine, and leukocyte signaling pathways. An example of the integration of multiple functional subpathways for one phase of an immune response (danger signal) is depicted in **Fig. 2**. The numbers of genes chosen in each subpathway are listed in **Table 4**, and the complete list of 1027 candidate genes and their primary subpathway (and secondary subpathway for a subset of genes) is provided in **Supplementary Table S1**.

**Table 3:** Pathways and proteins associated with immune response phases.

Phase of immune response	Examples of pathways, proteins, and inflammatory mediators involved in immune response phases
Hematopoiesis/homeostasis/ tolerance	hematopoietic cytokines (M-,G-,GM-CSF;IL-4,-5,-7,-13), stromal factors (c-kit, SCF, Flt3L), regulatory T cell function (Foxp3)
Danger signal	innate pathogen recognition receptors (TLRs, CARDs/NODs, peptidoglycan recognition proteins), scavenger receptors (MSR1), endothelins, adenosine receptors, complement, stress-induced responses (MIC-A,-B), eicosanoid synthesis genes, cytokines, antigen presentation genes
Mobilization of immune cells	systemic inflammatory mediators (IL-1β, IL-6, TNF-a), chemokines, eicosanoids, GPCR signaling (eicosanoids, histamines)
Extravasation	adhesion molecules (integrins, -CAMs), chemokines, vasodilators (eicosanoids/GPCR), cytoskeletal rearrangement singaling molecules (Vav, VASP, MENA), non-muscle myosins
Migration to site of inflammation	adhesion molecules (integrins, -CAMs, maxtrix receptors), chemokines, matrix proteases (MMPs), cytoskeletal rearrangement singaling molecules (Vav,VASP,MENA), focal adhesion proteins (Vav,ROCK), non-muscle myosins
Interactions between resident cells, immune cells, and pathogens at site of inflammation	adhesion molecules, innate detectors of pathogens (TLRs, CARDs/NODs), Fc receptors (FcgRI,II,III; FceRI,II), stress-induced ligands (MIC-A,-B), NK cell-activating receptors, cytokines and receptors, other activating receptors (TCR, BCR complexes; growth factor receptors); co-stimulatory receptors (B7 family, CD2 family), inhibitory receptors (KIRs, LIRs/ILTs), phagocytosis/antigen presentation (XBOX genes, CIITA, TAP, immunoproteasome, HLA molecules)
Activation of inflammatory cells	MAPK pathways (Erk, p38, Jnk), PI3K/Akt signaling, NF-kB signaling, cytokine signaling (JAK/STAT/Tyk, NFIL3, NFIL6, IRFs), GPCR signaling (PKA, PLCb, phosphodiesterases, CREB, Pyk2, Rap1, Src), adaptor signaling proteins (TRAFs, IRAKs, MyD88, DAP10, DAP12, ZAP70, Syk, LAT, SLP76, MyD88, CD3ζ, FcεRγ)
Effector functions of inflammatory cells	cytokines (IFN-γ, IFN-α, TNF-α superfamily, CSFs, interleukins), death receptor ligands (FasL, TRAIL, TNF-a), eicosanoids (prostaglandins, thromboxane, prostacyclin, leukotrienes), cytotoxic mediators (glutathiones/PHOX/reactive oxygen species, RNS, perforin/granzymes), antibody production, acute phase/fever response (C-reactive protein, factor P)
Response of target cells	cytokine receptors, GPCRs, death receptors, apoptosis signaling, adhesion molecules, growth factor receptors
Resolution of immune response vs. chronic inflammation	apoptosis (death receptor and mitochondrial pathways), TGF-β, IL-10, Foxp3, prostaglandins, phosphatases, inhibitors of cytokine signaling (SOCS, A20/TNFAIP3)

**Table 4:** Primary subpathways in inflammation panel.

Subpathway	Number of genes in subpathway	Number of SNPs in subpathway
Adhesion-Extravasation-Migration	142	1385
Apoptosis Signaling	68	682
Calcium Signaling	14	409
Complement Cascase	40	419
Cytokine signaling	172	1598
Eicosanoid Signaling	39	374
Glucocorticoid/PPAR signaling	21	230
G-Protein Coupled Receptor Signaling	42	1125
Innate pathogen detection	50	457
Leukocyte signaling	121	1743
MAPK signaling	118	1949
Natural Killer Cell Signaling	31	259
NF-kB signaling	33	297
Phagocytosis-Ag presentation	39	286
PI3K/AKT Signaling	37	307
ROS/Glutathione/Cytotoxic granules	22	162
TNF Superfamily Signaling	38	328

### Example application: WFINFLAM tSNP panel

Because the components comprising inflammation are very numerous and interacting across many pathways, without strong *a priori* evidence it is difficult to choose a handful of candidate genes to fully cover the potential genetic risk factors contributing to the inflammatory component of a particular disease. For this reason, panels of single nucleotide polymorphisms (SNPs) in an array of inflammation-related genes broadly covering most aspects of immunity and inflammation based on our assembled list will be critical in objectively evaluating the impact of genetic variations in inflammation-related genes on an inflammation-dependent outcome.

There is a commercially available product, Affymetrix GeneChip® Human Immune and Inflammation 9K SNP panel, that attempts to serve the purpose. This application -specific panel contains ∼9,000 SNPs to cover ∼1,000 immunity- and inflammation- related genes (http://www.affymetrix.com/support/technical/datasheets/humanimmune_9k_snp_datasheet.pdf). However, the rationale for choosing the ∼1,000 immunity and inflammation genes in this panel is not clear, and the coverage of these genes, regardless of their biological functions, may not be sufficient. In order to investigate the impact of genetic variations in a broad array of inflammation-related genes on disease risk, we created two tagging SNP (tSNP) panels, one each for populations with either Caucasian or African ancestries, for Affimetrix ParAllele genotyping chips. These tSNP panels were designed to capture majority of the genetic variations in these 1027 inflammation candidate genes.

tSNPs for the 1027 inflammation-associated candidate genes were chosen based on a pair-wise r^2^ threshold of 0.8 and MAF ≥5% using data in the HapMap Phase II database (HapMap Data Release 21a/phaseII; http://hapmap.org/index.html.en). tSNPs were chosen separately for the CEU (representing European ancestry) and YRI populations (representing African ancestry). In order to accommodate as many relevant inflammation genes as possible, less stringent criteria with r^2^ threshold >0.5 were employed for intronic regions (excluding 5kb in the beginning and end of these big introns) greater than 50kb in certain large genes with >100 tSNPs. There were seven genes in this category, and the intronic regions for which a less stringent r^2^ threshold was applied are listed in **Supplementary Table S2**. For genes without genotype information from HapMap, additional SNPs in six inflammation genes were chosen to be included based on information from other resources (see **Supplementary Table S3**), as described in *Methods* section.

The resulting inflammation tSNP panels, WFINFLAM-CEU for Caucasians and WFINFLAM-YRI for African descent, include 12,011 SNPs and 21,542 SNPs respectively in 1027 inflammation-associated candidate genes. There is an average of 11.7 and 21 SNPs in each candidate gene in WFINFLAM-CEU and WFINFLAM-YRI panels, respectively. **Table 4** briefly summarizes the numbers of genes and tSNPs included in each subpathway. The annotation table for this panel, including the list of 1027 genes, their chromosomal positions, their associated primary and secondary subpathways, and the number of tSNPs chosen for each gene can be found in **Supplementary Table S1** (and also our website: http://www1.wfubmc.edu/Genomics/PublicationsandData/). Additionally, the annotation file for the SNPs included in the WFINFLAM-CEU and WFINFLAM-YRI panels, including the chromosomal locations, their associated genes and the sub-pathways, can also be found in **Supplementary Tables S4 and S5** (and our website: http://www1.wfubmc.edu/Genomics/PublicationsandData/). The coverage for the 1027 inflammation candidate genes in CEU is better compared to other widely used genome-wide association panels. For the coverage of the 1027 inflammation-associated candidate genes, 90.4% of the genes have 90% or more SNPs within these genes that can be captured by r^2^≥0.8 in CEU using WFINFLAM-CEU panel, compared to 78.9% for the Illumina HumanHap 550 genome-wide panel and 45.8% for the Affymetrix 500k genome-wide panel. For populations with African ancestry, the coverage for the 1027 inflammation candidate genes in YRI is greatly improved compared to other widely used genome-wide association panels. For the coverage of the 1027 inflammation-associated candidate genes, 88.2% of the genes have 90% or more SNPs within these genes that can be captured by r^2^≥0.8 in YRI using WFINFLAM-YRI panel, compared to 27.1% for the Illumina HumanHap 650k genome-wide panel (which was designed to capture more genetic information from YRI population), and 12.5% for the Affymetrix 500k genome-wide panel. The coverage for all these genotyping panels is detailed in **Supplementary Table S6**.

## Discussion

Various components and complex interactions comprise immune and inflammation responses, and numerous genes are involved in this complex network. With a thorough review of various aspects of inflammatory immune responses, and a systematic search for gene-gene interactions using Ingenuity Pathway Analysis, we have provided a comprehensive list of inflammation-associated genes and subpathways for genetic association studies.

Genome-wide association studies have been a very popular approach to test the association between disease phenotypes and genetic variations. However, we believe there are still several advantages for a pathway-focused study. First of all, compared to whole-genome analyses, restricting analyses to SNPs in a specific pathway reduces the number of multiple tests performed in the analysis of a study population, thereby reducing the probability of false positive associations and increasing the effective power of the study. This kind of study design is particularly effective when inflammation plays an important role in disease etiology and the goal of the studies is to delineate genetic variations in inflammation pathway to disease risk and/or progression. A related second advantage of restricted pathway analysis is in study design. A large proportion of investigators may not have access to the very large number of subjects and multiple confirmation populations needed to overcome false positive associations due to multiple testing in genome-wide association studies. Studies restricted to a pathway analysis permit the use of study populations that are not large enough for use in whole-genome association studies. When target diseases are not prevalent and inflammation is obviously involved in disease etiology, researchers will gain the most out of an inflammation pathway-specific study design. Although some genes not related to inflammation found in whole-genome panels may impart some risk to inflammation-associated diseases, associated genetic variants would be anticipated to be concentrated in a panel of SNPs in inflammation-associated genes. Therefore, the drawback of potentially missing associated non-inflammation genes is offset by the increased probability of detecting true associations in an inflammation-restricted panel. Thirdly, pathway analysis is far less expensive to perform than whole-genome analysis, especially considering the cost for second, and/or third stage confirmation studies needed to follow-up the significant results from an initial screening in order to rule out false positive associations. Lastly, the functional subpathways are also pre-defined with available biological information. This refined information provides investigators with the opportunity to test gene-gene interactions within subpathways in which synergistic interactions are more likely to be concentrated. Additionally, the interplays between subpathways are also clearly defined to enable investigators to test biologically feasible interactions between subpathways.

However, the results from this manuscript also have potential utility for investigators who have more interests in surveying the whole genome. For whole-genome analyses where there is a prior hypothesis for inflammation being associated with the outcome, the inflammation pathway and subpathways defined in this manuscript may provide a framework for testing whether SNPs in the inflammation pathway or subpathways as a whole are overrepresented for significant associations to the outcome. Although pathway networks can be constructed for whole-genome analyses, such networks should be designed *a priori* before beginning the study.

The WFINFLAM and WFINFLAM-YRI SNP array panels for inflammation-associated genes provide a powerful tool for analyzing the contribution of genetic variation in diseases that have inflammatory components. Although whole-genome SNP panels are currently available that include almost all of the genes included in the WFINFLAM panels, the coverage of SNPs in genes included in the WFINFLAM array is superior to the coverage of currently available in whole-genome arrays, especially for populations with African ancestry background. For researchers who would like to use other genotyping platforms, the inflammatory gene list provided here would be a good starting point for designing genotyping assays for other platforms. Additionally, alternate approaches, other than r^2^ based method, for choosing SNPs based on the inflammatory gene list provided here could also be considered. For example, researchers may specifically focus on “high-prior” polymorphisms that are known to be functional or have been previously linked to the specific diseases under study, alone or in combination with the tSNPs provided in the WFINFLAM panel. This approach may be more efficient and powerful than the r^2^ based method alone, especially if the targeted “high-prior” polymorphisms are causal and their linkage to the nearby tSNPs is incomplete.

In addition, precaution may be warranted for tSNP panels designed based on the HapMap project. The transferability of the LD patterns between populations studied in HapMap project and other study populations may need to be validated. The transferability of HapMap-based selection of tSNPs using the reference CEU population to several other diverse populations of European ancestry has been demonstrated to be almost as effective for overall SNP coverage in selected genomic regions or randomly selected SNPs in the respective populations [46]–[50]. However, the coverage with tSNPs based on HapMap CEU data for a small subset of specific genes or SNPs may be lower in certain populations despite overall similar coverage [51], particularly for isolated indigenous populations [52]. Other than the data provided by the HapMap project, we are not aware of complete sequence variant information available for validation of the transferability of tSNP panels that were designed based on HapMap data to other study populations.

In summary, pathway analysis of inflammation-associated genes is a powerful approach for determining genetic risk factors for both inflammatory diseases and other diseases that may have an under-appreciated modest inflammatory component, such as cancers. The inflammation pathway gene list and functionally-defined subpathways provide useful tools for assessing the impact of genetic variations in inflammation pathways on disease risk, in situations where either pathway-focused studies or genome-wide analyses are employed.

## Methods

### Selection of genes

Networks of genes involved in the regulation of the phases of immune responses (described in *Results* and **Table 2** ) were built using Ingenuity Pathways Analysis (Ingenuity Systems: www.analysis.ingenuity.com). Both pre-defined ‘canonical pathways’ and custom-built pathways based on our own queries for genes/pathways not included in the canonical pathways were used to establish these networks. Canonical pathways included: actin cytoskeleton signaling; antigen presentation pathway; apoptosis signaling; B cell receptor signaling; calcium signaling; cAMP signaling; chemokine signaling; complement and coagulation cascade; death receptor signaling; ERK/MAPK signaling; FcEpsilon receptor signaling; G-coupled protein receptor signaling; GM-CSF signaling; IGF-1 signaling; IL-10 signaling; IL-2 signaling; IL-4 signaling; IL-6 signaling; integrin signaling; interferon signaling; JAK/STAT signaling; leukocyte extravasation signaling; natural killer cell signaling; NF-kB signaling; nitric oxide signaling; notch signaling; p38 MAPK signaling; PI3K/Akt signaling; PPAR signaling; protein ubiquination pathway; PTEN signaling; SPK/JNK signaling; T cell receptor signaling; TGF-β signaling; Toll-like receptor signaling.

These networks were then arranged into inflammation subpathways by:

1. combining several networks together (e.g., ERK/MAPK, p38 MAPK, and SAPK/JNK canonical pathways into the ‘MAPK signaling’ inflammation subpathway; IL-2-, IL-4-, IL-6-, IL-10-, Interferon-, GM-CSF-, IGF-1-, JAK/STAT6-, and TGF-β- signaling canonical pathways into ‘cytokine signaling’ inflammation subpathway; actin cytoskeleton-, chemokine-, integrin-, and leukocyte extravasation- signaling canonincal pathways into the ‘adhesion-extravasation-migration’ inflammation subpathway; etc.);
2. adding additional genes to bridge networks within a subpathway and to include appropriate genes not included in the canonical pathways (e.g., additional cytokines and their receptors were added to the ‘cytokine signaling’ inflammation subpathway; additional integrins and chemokines/chemoattractant molecules were added to the ‘adhesion-extravasation-migration’ inflammation subpathway; CD antigens expressed by leukocytes not already included were considered for addition to several subpathways; other missing genes/pathways considered important by the panel of investigators, such as the scavenger receptor network for ‘leukocyte signaling’ inflammation subpathway and Nod1/CARD family networks for ‘innate pathogen detection’ inflammation subpathway; etc.);
3. trimming the networks of genes with low priority for inclusion in the inflammation panel. Genes with lower priority include: genes not expressed in immune cells or not directly involved in cells responding to inflammation, including non-immune cells (e.g., skeletal muscle-specific myosins in the actin cytoskeleton signaling canonical pathway; calsequestrins expressed mainly in various muscle cells, in the calcium signaling canonical pathway; GH1 and GHR, growth hormone expressed in pituitary gland and its receptor, and NGFB and NGFR, nerve growth factor and its receptor, in the NF-κB canonical pathway; etc.; MAPK8IP1, specific for pancreatic cell function, in the PI3K/AKT canonical pathway; etc.); genes with unknown function, though genes with high homology to known inflammatory mediators were considered (e.g., bcl-2 family homologs, IL-1β family homologs). Special emphasis was placed on genes at nodes for signaling to and from multiple pathways, most notably genes in NF-κB, MAPK, and PI3K signaling pathways.

### Choosing tagging SNPs

Tagging SNPs (tSNPs) for candidate genes were chosen using Tagger server (http://www.broad.mit.edu/mpg/tagger/server.html). The target sequences included genomic regions containing the entire candidate genes, 5kb before transcription start site, and 2kb after the transcription end site, based on annotation in NCBI Build 35. When candidate regions for two or more genes overlapped, the combined genomic regions were used for choosing tSNPs. Two separate lists of non-synonymous coding SNPs for the CEPH population (CEU) and for the Yuruba population (YRI) were downloaded from HapMap (rel21a_NCBI_Build35) and forced in as tagging SNPs for ancestry-specific panels. Pair-wise r^2^ threshold of 0.8 and MAF≥5% were used. For genes without genotype information from HapMap, SNPs in Affymetrix 500k array, as well as SNPs with frequency data from the Innate Immunity PGA (IIPGA) (http://innateimmunity.net/), were manually chosen to be included in the list based on allele frequencies and inter-SNP distance (the chromosomal positions are based on IIPGA annotation because some of these genes/SNPs were ambiguously mapped in build 35). Some tSNPs were dropped out from the final list because they did not pass the Affymetrix design review due to the following reasons: non-biallelic SNPs, existing SNPs or ambiguous bases too close to the SNP of interest, SNPs exceeding T_m_ ranges, or SNPs failing BLAST searches. In the attempt to fill in the gaps due to tSNPs failing design criteria, tSNPs for genes with dropped-out tSNPs were chosen again by forcing out the tSNPs failing design review and forcing in the remaining tSNPs when running Tagger. In total, there are 12,011 SNPs in WFINFLAM panel for Caucasians and 21,542 SNPs in WFINFLAM-YRI for African descents in 1027 inflammation-associated candidate genes that passed the Affymetrix design review.

### Estimation of genomic coverage by tagging SNPs

We used LdCompare (Hao 2006; http://www.affymetrix.com/support/developer/tools/devnettools.affx) to estimate the coverage of the 1027 inflammation-associated candidate genes with both WFINFLAM panel for Caucasians and WFINFLAM-YRI panel for African descents. We have also computed the coverage of several commercial genotyping arrays, including Affymetrix Mapping 500K, Illumina HumanHap 550 and HumanHap 650, for these 1027 genes. Single-marker coverage (pairwise r^2^) and multiple-marker coverage (multiple-marker r^2^) were computed and combined to estimate the coverage for the genomic regions containing the entire candidate genes, 5kb before transcription start site, and 2kb after the transcription end site. The summary for the coverage for these genes in all these genotyping panels are detailed in **Table S6**.

## Supporting Information

Table S1 List of genes included in WFINFLAM and their associated sub-pathways
(0.19 MB XLS)

Table S2 Genes with big intronic regions
(0.06 MB XLS)

Table S3 SNPs chosen for the six genes without HapMap genotyping data
(0.04 MB XLS)

Table S4 Annotation for tSNPs in WFINFLAM_CEU panel
(1.64 MB XLS)

Table S5 Annotation for tSNPs in WFINFLAM_YRI panel
(3.39 MB XLS)

Table S6 Summary for gene coverage of Wfinflam panels and other genome wide association panels
(0.04 MB XLS)
